# Nanodiamonds for device applications: An investigation of the properties of boron-doped detonation nanodiamonds

**DOI:** 10.1038/s41598-018-21670-w

**Published:** 2018-02-19

**Authors:** Abdulkareem Afandi, Ashley Howkins, Ian W. Boyd, Richard B. Jackman

**Affiliations:** 1 London Centre for Nanotechnology and the Department of Electronic and Electrical Engineering, University College London, 17-19 Gordon Street, London, WC1H 0AH UK; 2 ETC, Bragg Building, Brunel University, Uxbridge, UB8 3PH UK

## Abstract

The inclusion of boron within nanodiamonds to create semiconducting properties would create a new class of applications in the field of nanodiamond electronics. Theoretical studies have differed in their conclusions as to whether nm-scale NDs would support a stable substitutional boron state, or whether such a state would be unstable, with boron instead aggregating or attaching to edge structures. In the present study detonation-derived NDs with purposefully added boron during the detonation process have been studied with a wide range of experimental techniques. The DNDs are of ~4 nm in size, and have been studied with CL, PL, Raman and IR spectroscopies, AFM and HR-TEM and electrically measured with impedance spectroscopy; it is apparent that the B-DNDs studied here do indeed support substitutional boron species and hence will be acting as semiconducting diamond nanoparticles. Evidence for moderate doping levels in some particles (~10^17^ B cm^−3^), is found alongside the observation that some particles are heavily doped (~10^20^ B cm^−3^) and likely to be quasi-metallic in character. The current study has therefore shown that substitutional boron doping in nm NDs is in fact possible, opening-up the path to a whole host of new applications for this interesting class of nano-particles.

## Introduction

Nanoscale diamonds (NDs) are finding increasing numbers of applications^1^ in fields as diverse as electronics^2^, spintronics^3^, electrochemistry^4^, biotechnology^5^ and medicine^6^, as well as being used as ‘seeds’ during chemical vapour deposition (CVD) of diamond films^7^. Diamond can be considered as a wide band gap semiconductor (E_g_ ~ 5.5 eV), which behaves as a dielectric unless purposefully doped. The preeminent technique for the production of NDs is a detonation process^8^ that typically involves detonating a mixture of TNT and hexogen in an oxygen-deficient environment^9^. The reaction conditions result in the formation of micron-sized aggregates of nanometre scale diamonds; techniques have been developed^10^ that enable de-aggregation resulting in individual NDs in the size range 3–10 nm. The surface of detonation synthesised nanodiamonds (DNDs) host a wide range of oxygen and hydrogen based moieties, and can be functionalised with a wide range of chemical groups^5^. Plasma-enhanced CVD grown diamond is often doped with boron, by the inclusion of boron containing gases to the growth plasma; boron imparts electrical conductivity from p-type character through to semi-metallic behaviour at high concentrations^11^, and is also associated with superconductivity within diamond films^12^. Boron-doped NDs (B-NDs) would be an attractive material for many applications including high surface area electrodes for electrochemistry, electrode material within supercapacitors, electro-catalytic material within fuel cells and for nm-scale diamond electronic devices and sensors. Moreover, the widespread use of undoped DNDs as seeds for the subsequent growth of boron-doped diamond films leads to the inevitable introduction of a highly resistive layer between the doped film and the substrate that is deleterious for many applications^13^; larger B-NDs (rather than nm-scale B-DNDs) have indeed been investigated by others to overcome this problem^13,14^, but the density of such seeds can never approach that of the density achieved from DNDs.

Attempts to generate boron-doped NDs to date have relied on either the growth of a boron-doped layer over undoped NDs^15^ or by solid-state diffusion^16^, but the remaining insulating diamond core leads to a high over all resistivity for the doped material. Heavily boron doped NDs have been produced using a milling process to crush boron-doped CVD grown diamond^14^, yielding NDs in the size range 10–60 nm. There are currently no reports of the properties of boron doped DNDs, whose ~5 nm size is essential for several applications, where boron-containing material has been purposefully added to the synthesis environment, although a process for doing this has been proposed by Shenderova^17^. One method involves the introduction of boron containing graphite or boron carbide powders to the combustion mixture prior to detonation. Theoretical predictions for the properties of boron containing NDs with dimensions typical for DNDs vary between the suggestion that they will primarily be located at the surface^18^, be metastable within the ND core and therefore unstable^19^ or form a stable substitutional state in the ND core^20^. This paper reports an experimental investigation into the properties of boron-doped DNDs giving evidence for substitutional doping.

**Figure 1:**
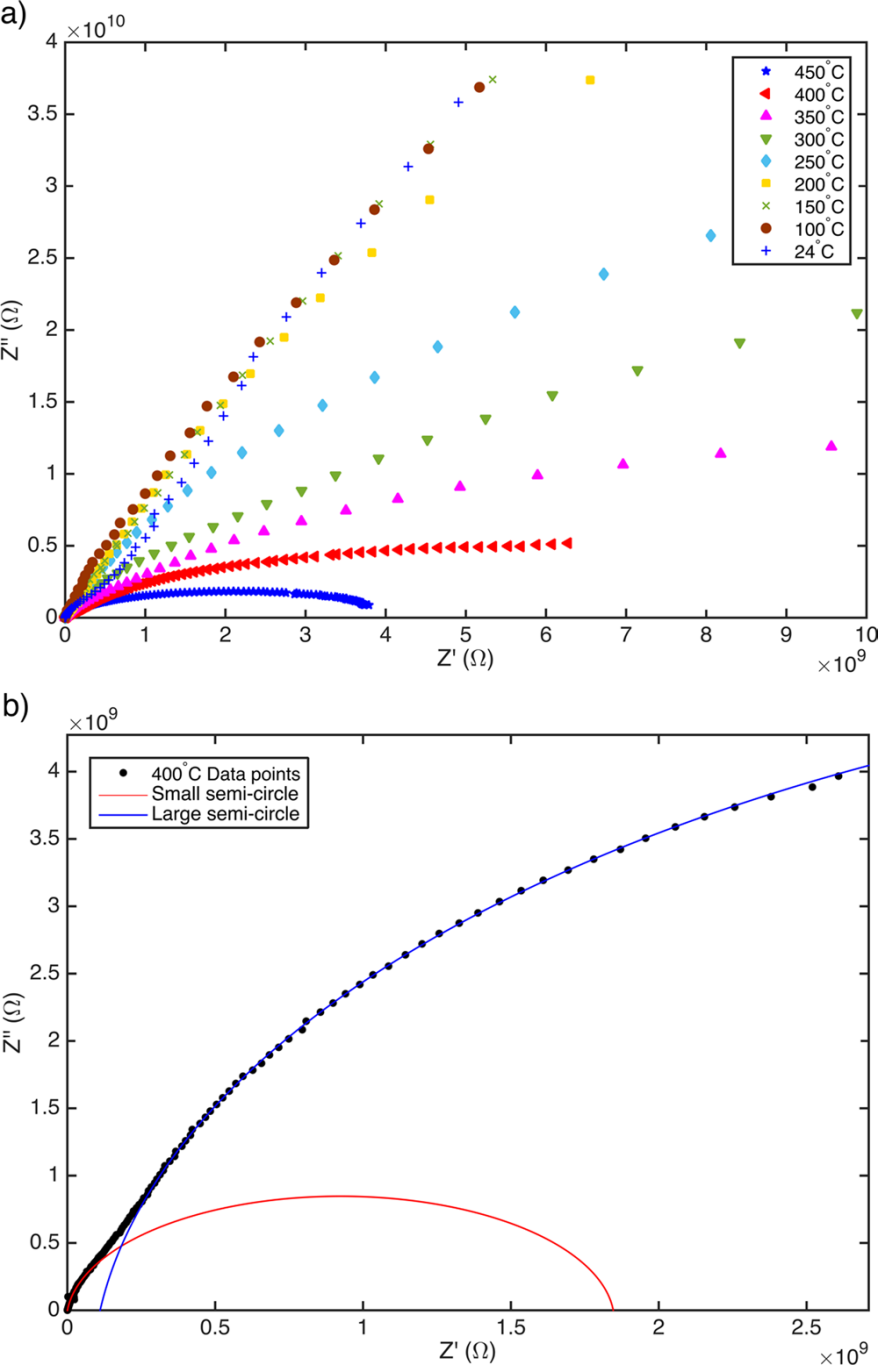
(**a**) Cole-Cole plot of impedance measurements between 50 °C and 450 °C, and (**b**) Two semi-circles fitted to Cole-Cole data at 400 °C.

**Figure 2:**
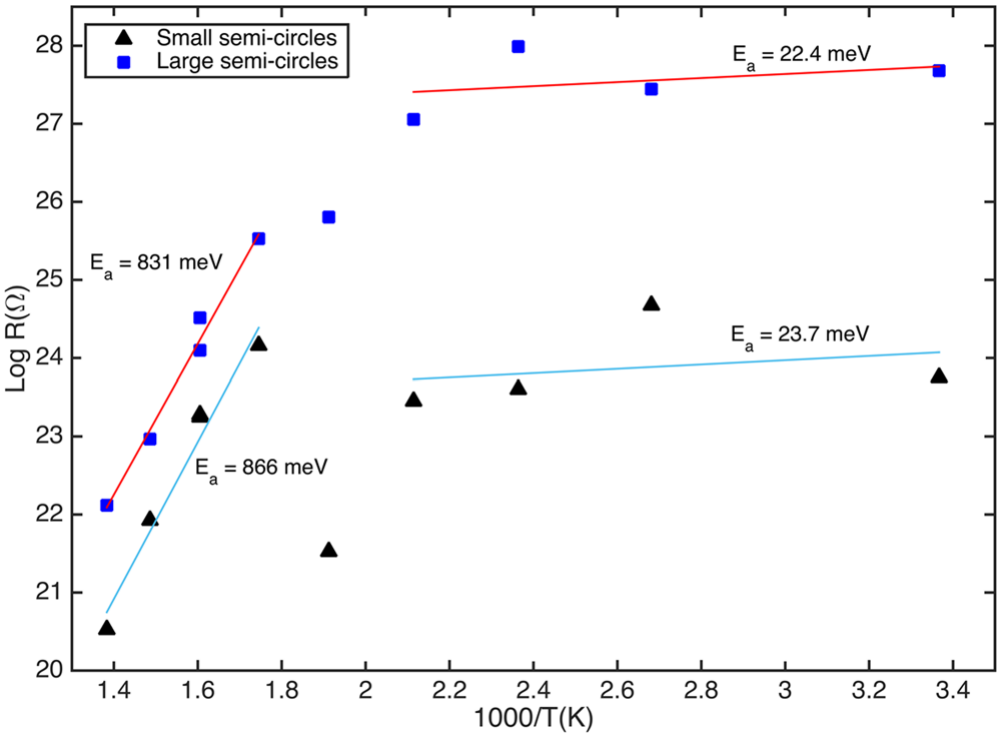
Arrhenius plot with linear fitting for the R-values determined from impedance measurements at differing temperatures.

B-DNDs were investigated using AC impedance spectroscopy (IS), which can give insight into the differing electrical conduction processes that are present^21^. Here the NDs were compacted and IS measurements taken in a ‘sandwich’ configuration. Figure 1(a) shows the real vs imaginary components plotted against each other as a function of measurement frequency – plots at several temperatures are included. These so-called ‘Cole-Cole’ plots should reveal a semicircular response for a given conduction path, which can be modelled as an RC circuit component^21^. It is clear from Fig. 1(a) that no single semicircular response is apparent. Using established methods for IS curve fitting^21^ individual semicircular responses can be determined from this data, as shown in Fig. 1(b) for a given temperature. Two distinct semicircular responses can be proposed, one at higher measurement frequencies than the other. R and C values can be determined from the fitted plots assuming two RC circuits exist (one per conduction path) in series. Arrhenius style plots, for the value of resistance (R) plotted as a function of temperature, enable determination of the thermal activation energy for each suggested conduction path to be determined, as shown in Fig. 2. For each of the two semicircles (large and small), two plateaus can be observed, each associated with a conduction path. This suggests the two different activation energies for the conduction processes are apparent; a higher activation energy of E_a_ = ~0.8 eV at temperatures between 300 °C and 450 °C, and a much lower activation energy E_b_ = ~0.02 eV below 200 °C.

**Figure 3:**
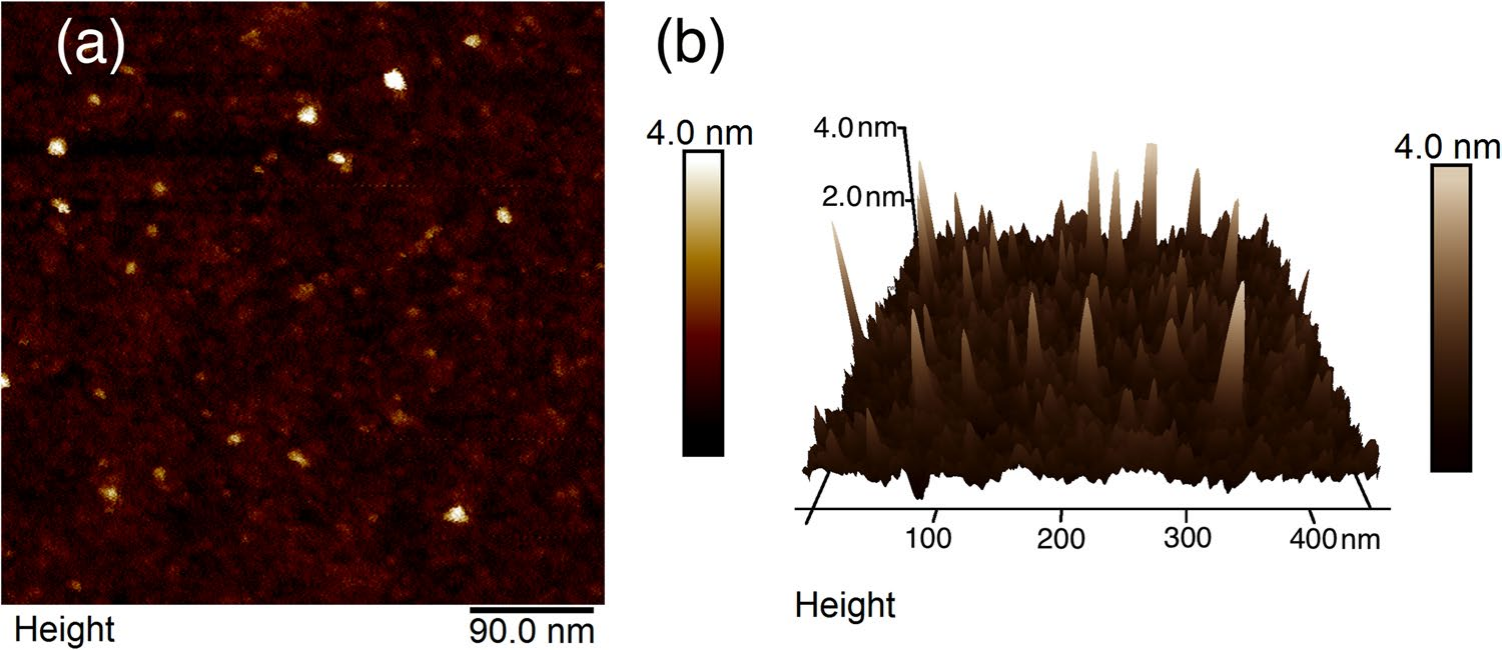
(**a**) Atomic force microscopy image of Boron-DND aggregates and individual DNDs on SiO_2_ and (**b**) 3D representation of this image.

Atomic force microscopy (AFM, tapping mode) was used to identify the average grain size of the DNDs. Using AFM in tapping mode, a laser excites the cantilever near its resonant frequency, and the tip will resonate. Depending on the atomic forces close to the surface, the cantilever’s oscillations will be affected by the van-der-Waals forces, which are dominant up to a few nanometres from the surface. A feedback system keeps the oscillations constant by varying the distance from the surface, hence allowing topographic imaging with sub-nanometre accuracy. This method should allow accurate measurements of heights of single DNDs, but not of their widths parallel to the substrate’s surface. However, given that DNDs are generally symmetric in shape (confirmed using HR-TEM, see below), height measurements should be sufficient to verify their average size. A typical AFM image of DND aggregates deposited onto a SiO_2_-Si substrate (ultra-sonication from an aqueous solution) is shown in Fig. 3; this image confirms that individual DNDs within the aggregates have diameters within the expected range.

**Figure 4:**
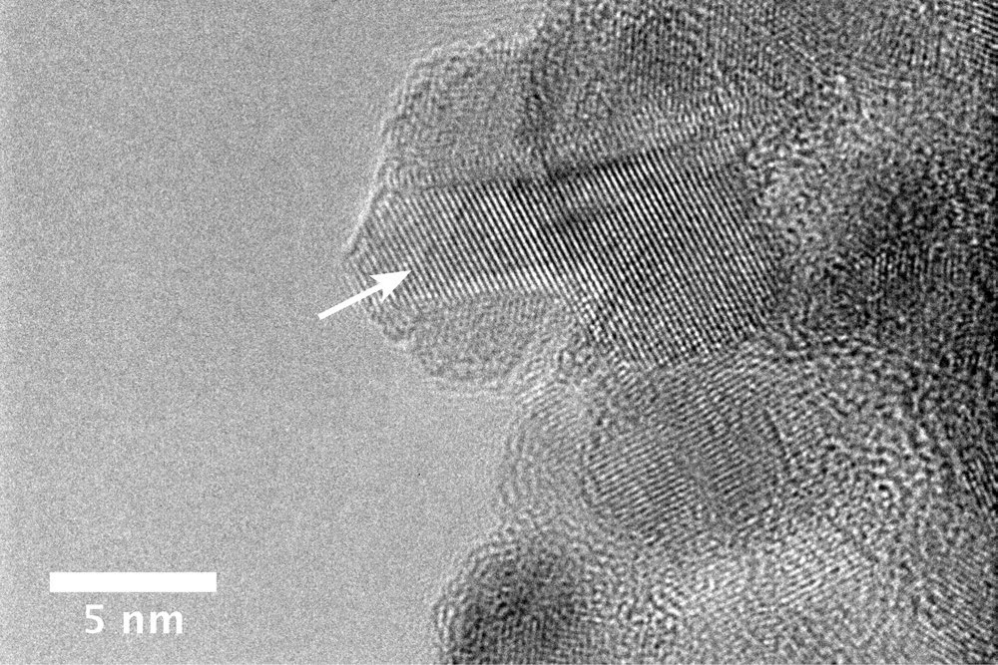
HR-TEM image of DNDs. The arrow is pointing at the (111) diamond lattice lines.

To verify the atomic structure of the DNDs, High resolution-transmission electron microscopy (HR-TEM) was used. HR-TEM images of the B-DNDs confirm the crystalline structure of the nanodiamonds, and clearly show diamond cores surrounded by sp^2^ carbon structures around the cores (Fig. 4). The presence of NDs is clear from the lattice spacing of (111) diamond faces (0.206 nm), shown by the arrow in the figure. Overlapping of DND crystal lattices can also be seen clearly. The lattice spacing increased to 0.213 nm in other locations, possibly indicating the presence of dopants such as Nitrogen and Boron.

**Figure 5:**
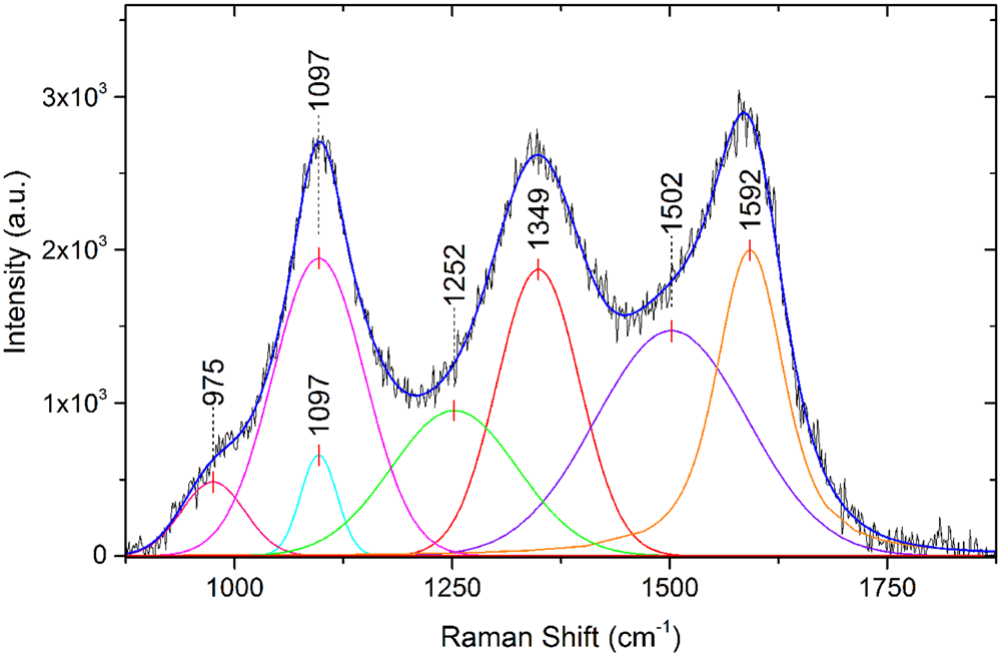
Raman spectrum of B-DNDs.

**Figure 6:**
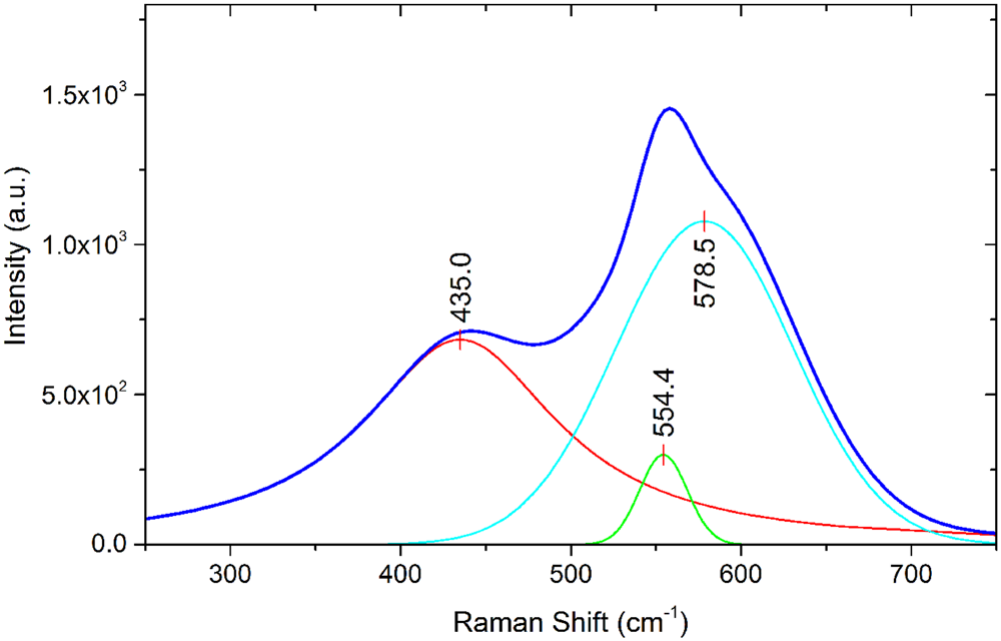
Fitted Raman spectrum of the 500 cm^−1^ peaks for the B-DNDs.

Raman spectroscopy is a non-destructive method, which can be used to detect carbon crystal structures and obtain information about their quality and composition, where vibrational modes in Raman active molecules scatter light. Using an excitation wavelength of 532 nm, the obtained spectrum is composed of modes corresponding to sp^3^ and sp^2^ bonded structures, as well as Boron content above a minimum concentration^22^. Typical results are shown in Fig. 5, following fitting and photoluminescence background subtraction^23,24^. Sample shows prominent features at Raman shifts of about ~1100 cm^−1^, ~1350 cm^−1^, and ~1590 cm^−1^; the peaks correspond to trans-polyacetylene in grain boundaries, the sp^2^ carbon D peak, and the sp^2^ carbon G peak respectively^25,26^. The diamond peak expected at ~1332 cm^−1^ in bulk material is expected to be upshifted and broadened for nm-scale diamond^23^. A detailed summary of the observed features and their fitting is in Table 1. Other peaks may also be present, such as the ~1200 cm^−1^ peak usually present in highly Boron-doped diamond films, which is usually accompanied by a 500 cm^−1^ peak (Fig. 6); the peaks centred around 500 cm^−1^ can be used to estimate the substitutional Boron concentration in diamond thin films^24^.

**Table 1:** Raman spectroscopy features of O-DND sample.

Peak Type	Centre (cm^−1^)	FWHM
Gaussian	975.2	86.1
Gaussian	1096.7	49.3
Gaussian	1096.8	125.0
Gaussian	1252.4	170.9
Voigt	1349.1	112.1
Voigt	1502.0	206.3
Voigt	1592.3	92.6

**Figure 7:**
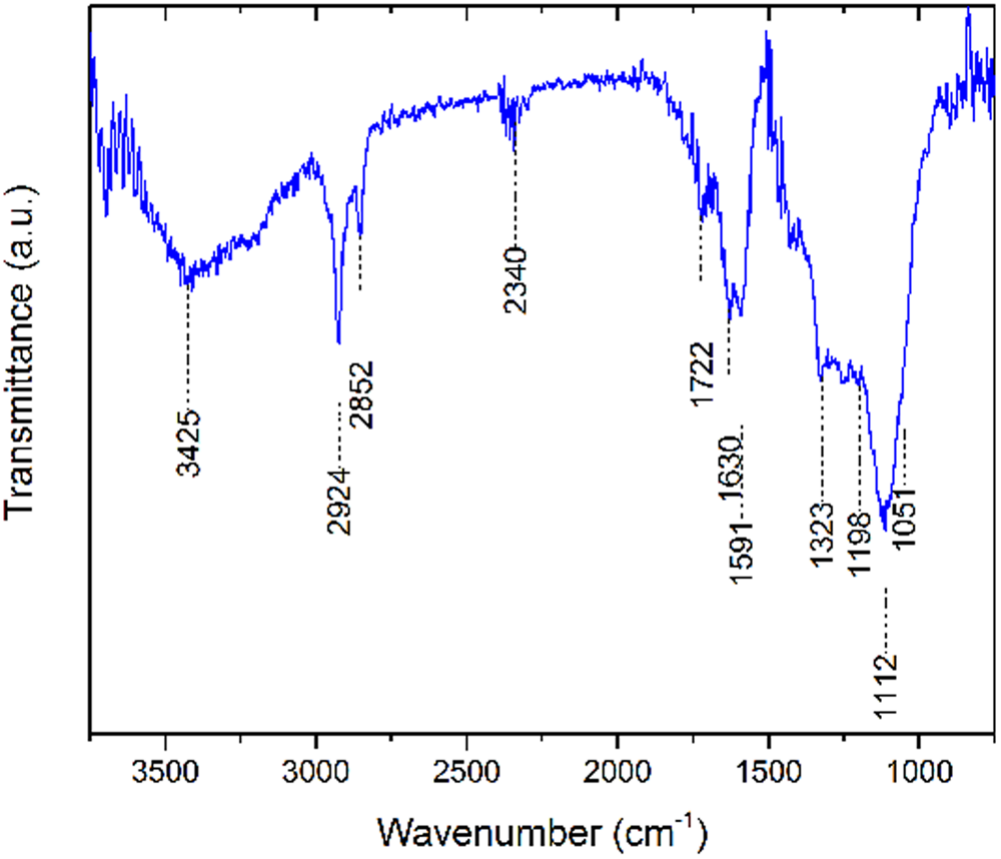
ATR spectrum of B-DND powder.

**Figure 8:**
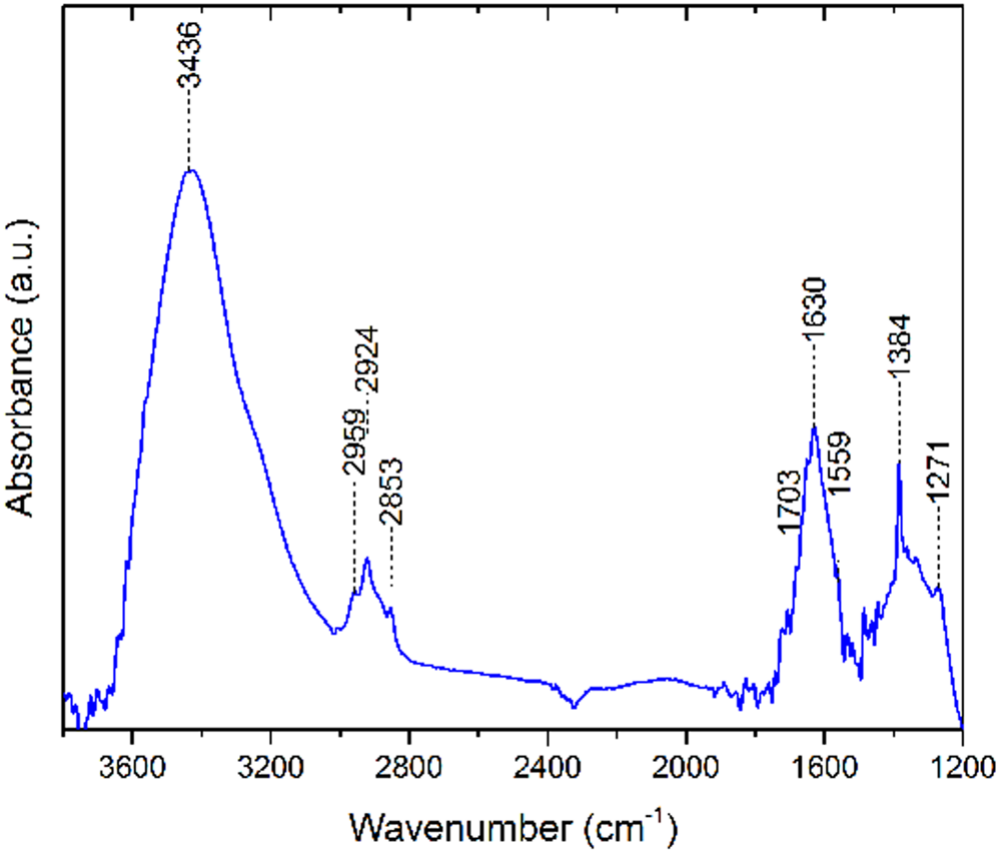
FT-IR spectrum of B-DNDs in a KBr pellet.

To obtain information on the surface composition of our DND samples, ATR and FT-IR spectroscopy, which can measure absorption of infrared light passing through vibrating molecules on the surface were performed. FT-IR measurements used DNDs mixed into KBr pellets, while ATR measurements were made by directly pressing DND powder on top of the ATR crystal. ATR measurements of the as-received DND powder show an abundance of organic groups on the surface (Fig. 7), including a probable signature of boric acid^27^. This is likely due to surface groups left from the detonation method used to produce the DNDs, and the subsequent (acid based) treatments typically used to purify the material recovered from the detonation chamber^28^. Prominent features in the FT-IR spectrum, shown in Fig. 8, are a peak at 1630 cm^−1^ on top of a 1550–1700 cm^−1^ broad feature, representing the C=C bond stretch, while a smaller feature from ~2850 to about 3000 cm^−1^ can be attributed to the C–H stretch. A large feature between 3000 and 3650 cm^−1^ is for the O–H stretch. The ATR spectrum looks similar to the FT-IR spectrum above ~1250 cm^−1^, but also includes a ~1050–1150 cm^−1^ C–O stretch peak on top of a broad feature, extending from between 1000 and 1500 cm^−1^, which could also include the signature for –C–H bending^29^.

**Figure 9:**
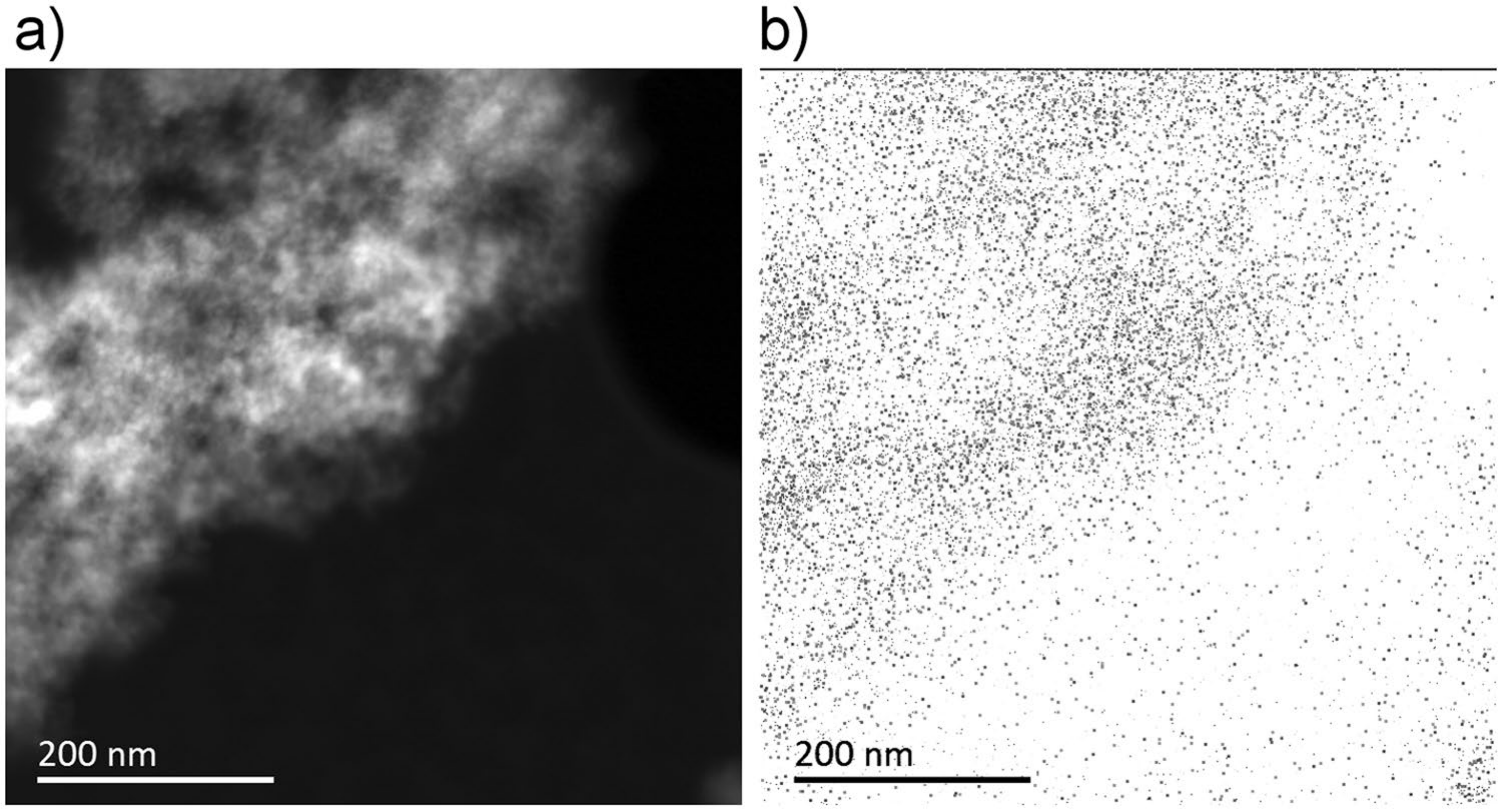
(**a**) Deep-field HR-TEM image of B-DND agglomerates and (**b**) corresponding cathodoluminescence obtained at 235 ± 5 nm wavelength.

**Figure 10:**
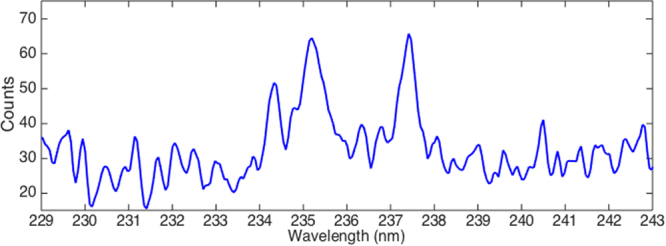
Cathodoluminescence spectrum of B-DNDs.

**Figure 11:**
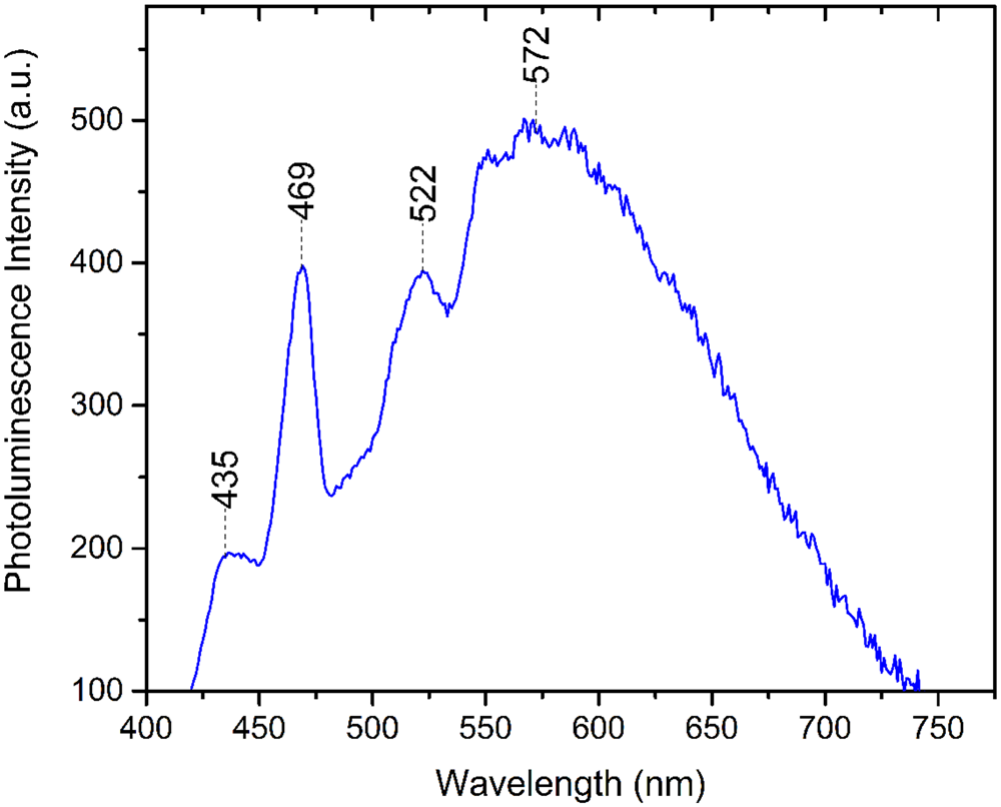
Photoluminescence spectrum of B-DNDs seeded on silicon.

Cathodoluminescence (CL) spectra of B-DNDs were taken at a low temperature (95 K) across aggregates, with an electron beam diameter of 20–200 nm. The electron beam provides enough energy to excite an electron to the conduction band, which leaves a hole that luminesces when filled by an electron. Spectra collected around several clusters of agglomerated DNDs showed differing relative sizes of peaks corresponding to both free excitons and boron bound excitons^30^. Scans were collected around 235 nm where excitonic features are expected. Figure 9(a) shows a HR-TEM image of agglomerated B-DNDs, with Fig. 9(b) showing the corresponding CL image (at 235 ± 5 nm) showing correlation between the two. The free excitons attributed to defects in diamond are expected to be centred ~235 nm compared to those of boron bound excitons at ~238nm^30^. Whilst this spectral resolution was not possible when using the microscope for imaging, de-focussing enabled the CL intensity to be collected over many aggregates with improved spectral resolution, as shown in Fig. 10. The ratio between the two clearly visible peaks is approximately 1. Another luminescence technique, photoluminescence (PL), was additionally used to measure the response of the DNDs to excitation. Unlike CL, PL measurements are limited by the photon energy of the source, and no features at higher energies can be detected. The PL spectrum obtained using a 405 nm laser shows the presence of three prominent peaks at 435, 469 and 522 nm, as shown in Fig. 11. The peak at 522 nm corresponds to energy of 2.39 eV. The features at wavelengths 435 and 522 nm do not directly correspond to the diamond free excitons and bound boron excitons. However, their presence in CVD diamond materials is usually accompanied by the presence of peaks around 575 and 636 nm, attributed to N-V centres. That these peaks are not seen here indicates that a significant amount of substitutional boron is present, filling up vacancies, thus preventing significant N-V centre formation^24^. The peak at 469 nm may be explained by the existence of N4 nitrogen centres, which are composed of four substitutional nitrogen atoms surrounding a single vacancy^31^.

Cathodoluminescence measurements have been used extensively to investigate the boron concentration in CVD grown diamond^30^. From the CL results presented here for B-DNDs it is found that the ratio of free excitons (FE)/ to bound excitons (BE) (5.27 and 5.21 eV respectively) is about 1 in most sampled locations. Taking the methodology used for CVD diamond then implies an average boron concentration of 1–2 × 10^17^ cm^−3^ to be present in the B-DNDs measured here^32^. Although this reasoning was developed for macroscopic CVD grown boron diamond, the fact that the B-DNDs here are agglomerated in clusters of hundreds of nm to a few microns will likely allow the existence of free excitonic features, regardless of the small size of individual DNDs. The result is temperature dependent^32^, and the spectra were obtained at approximately 95 K. It is important to note that edge emission (free excitons) are expected from (100) growth segments, but not in (111), where impurities like boron and nitrogen are more likely to exist^31,32^. CL measurements performed here contained contributions from many clusters of B-DNDs given that the electron beam has a diameter of around 200 nm, making the simultaneous observation of emissions from different growth sectors likely. The photoluminescence (PL) feature with the peak at 469 may be explained by the existence of N4 nitrogen centres, which are composed of four substitutional nitrogen atoms surrounding a vacancy^31^. According to Collins, there is no evidence that this form of nitrogen impurity behaves as a donor in diamond. The 522 nm peak is assigned to the first order (Raman) peak in PL. In lightly boron doped diamond, the first-order Raman peak is usually accompanied by an N-V centre luminescence peak at 636 nm. The suppression of this 636 nm peak in the PL spectrum, has previously been attributed to an increase of boron in the diamond lattice, potentially saturating vacancies in N-V centres^33^. The 575 nm N-V centre peak is also missing from the B-DND PL spectra recorded here, offering further evidence for the presence of substitutional boron.

The Raman spectrum obtained from B-DND powder confirms the presence of detonation nanodiamonds^26^, although the peak associated with bulk diamond (1332 cm^−1^) was not clearly seen, rather a broad peak centred around 1349 cm^−1^. According to a number of authors, the 1332 cm^−1^ diamond Raman peak shouldn’t be detected for NDs smaller than 5 nm^26,34,35^. The rest of the spectrum is mainly composed of two further peaks on top of a broad feature between ~1000 and 1600 cm^−1^. Carbon sp^2^ bonds are detected in the form of D (~1345 cm^−1^) and G (~1580 cm^−1^) peaks^26^. The near equivalence of the intensity of the three main peaks suggests an sp^2^ content of between ~1–2% of total bonds. This is due to the fact that sp^2^ carbon bonds have a significantly higher Raman efficiency when compared to sp^3^ ^36^. The ratio of the D to G peaks increases with decreasing crystal size of graphitic structures^36^. An additional peak in the Raman spectrum around 560 cm^−1^ was observed. This could be attributed to the transverse acoustic one-phonon mode seen in nanodiamond-films with sp^3^ bonded grains smaller than 5 nm^37^. However, this feature clearly comprises more than one peak, suggesting the boron doping-related feature around ~500 cm^−1^ may also be present. Heavily boron doped diamond films are known to have features at ~500 cm^−1^ and ~1220 cm^−1^. A Lorentzian component of a peak between 460 and 500 cm^−1^ shifts to lower wavenumbers as boron content increases, while a Gaussian component between 500 and 550 cm^−1^ can also be fitted, according to May^24^. If the 435 cm^−1^ peak is taken to be the Lorentzian component, then a boron concentration of ~7 × 10^21^ is calculated using Eqn. 1 of May^24^. However, if the Lorentzian is taken to be the peak at ~554 cm^−1^, then a boron concentration of ~2 × 10^19^ can be determined using the same equation. The 1100 cm^−1^ peak can shift depending on exciting wavelength, and is around 1100 for a 514 nm laser^26^. According to Ferrari, the 1100 cm^−1^ peak is due to trans-polyacetylene at grain boundary^25,26^. The ~1345 cm^−1^ Raman scattering peak may be attributed to both sp^2^ amorphous carbon (D peak) and diamond (1332 cm^−1^)^23^. A diamond peak can become broad due to the presence of impurities, in addition to strong phonon scattering from NDs^20^. Using visible excitation, the ~500 and ~1220 cm^−1^ features are detected in highly boron doped diamond films^24^. Although 500 cm^−1^ features are clearly seen, a 1220 cm^−1^ feature is not clearly obvious, although it could be hidden within the broad feature between 1000 and 1600 cm^−1^.

The presence of Boron and other dopants in ND cores of some of the nanodiamonds is inferred from the longer C-C bond spacing seen in NDs^38^, where the bond length increased by about 5 pm. This can also indicate other dopants (N being most likely candidate). Although nanodiamonds are clearly visible in HR-TEM imaging, the distribution of dopants cannot be directly inferred, due to its limited field of view. However, the fact that some regions display the expected longer C-C bond length supports the assertion that at least some of the NDs may have substitutional boron present. Shells surrounding NDs, composed of sp^2^ structures, are also seen surrounding diamond cores in HR-TEM images.

In the case of IR spectroscopy, Jiang *et al*. argue that phonon confinement due to the size of individual NDs (<5 nm) hinder the two-phonon process in IR between 1900 and 2500 cm^−1^. The same confinement effect is also partly responsible for the missing 1332 cm^−1^ Raman peak in small NDs^34^. FT-IR spectra of B-DNDs above 1500 cm^−1^ are nearly identical to spectra obtained from undoped NDs, suggesting similar surface terminations created by the detonation and subsequent cleaning process. The boron characteristic absorption IR line in diamond, at ~1290 cm^−1^, can be used to estimate the boron content of diamond films^39^. For a 1 mm thick KBr pellet mixed with Boron DNDs (~10:1 by weight), absorbance of 3% gives a boron content of ~6 × 10^17^ cm^−3^, a result close to the estimate from CL peak analysis.

In previous work, aggregated detonation nanodiamonds, without boron doping, have been shown to display only a single semicircular response in Cole-Cole plots determined from impedance spectroscopy^40^. Indeed, the material showed near-to ideal dielectric behaviour. Similar measurements on mono-dispersed DNDs revealed similar properties^41^. Therefore, whilst the presence of two conduction paths, indicated from the two semicircular responses in the impedance spectroscopy data (Fig. 1) could be discussed in terms of diamond grain and diamond grain boundary conduction seen in intrinsic^42^ and boron containing^43^ nanocrystalline diamond *films* this is not a likely explanation here. At low temperatures both conduction paths display low activation energies (20–30 meV); whilst moderate boron doping in thin film diamond is associated by an activation energy of 0.37 eV, this value decreases to meV values as the boron doping concentration increases^11^. Given the TEM observations (some grain doping, some grains with graphitic outershells), with the suggestion from the Raman that at least some material is heavily doped and there is graphitic material present, then the most likely attribution for the two conduction paths at the lower temperatures is due to these two materials-phases. Given one of these is attributed to boron doped DNDs, the activation energy of ~22 meV translates to ~1 × 10^20^ cm^−3^ boron content when applying the formula developed by Borst and Weiss^11^. That CL and PL suggest the presence of lower B-doping would be consistent with these particular grains contributing little to the conduction observed in IS due to their inherently higher activation energy. That the CL and PL techniques do not indicate heavily doped material is not surprising as at high boron levels, B no longer acts as a discrete centre but rather forms an impurity band^11^ which will not contribute to sharp features in these excitation measurements. The Arrhenius plots (Fig. 2) show that at higher temperatures an increased activation energy (~0.8 eV) is apparent; this has been previously observed in a number of diamond studies, and can be attributed to conduction from deep traps arising from defects within the diamond^42^.

When the CL, PL, Raman, FTIR and IS observations are combined it can be concluded that the B-DNDs studied here comprise a mixture of grains with varying boron content, likely with un-doped DNDs. The observation of bound excitons is also suggestive for stable substitutional boron doping (although other effects can also cause this). When the information from all techniques is considered together it is clearly indicated that DNDs with boron concentrations in the 10^17^ cm^−3^ range exist suggest, and that these NDs will therefore act as semiconductors. In addition, TEM observations of some grains indicated enlarged lattice spacing. This observation supports the theoretical predictions of Barnard and Sternberg^20^, but disagree with some others. That a conduction path most likely attributed to a highly boron doped ND, also suggested by Raman measurements, suggest other grains display quasi-metallic behaviour typical of diamond with a boron impurity band^11^, which again supports the assertion that NDs with a size <5 nm are capable of supporting substitutional boron, rather than it forming clusters or simply existing on grain edges^18,19^.

Nanodiamonds are an important class of nanocarbons for many applications, with NDs produced by a detonation process being unique in offering access to the sub-5nm particle size. The inclusion of boron within nanodiamonds to create semiconducting properties would create a new class of applications in the field of nanodiamond electronics, but to date examples of boron doped NDs are principally limited to ‘crushed’ and milled CVD diamond films^17^, such materials do not offer low-nm sizes and the properties that are associated with this scale, although a recent report on a high-pressure high temperature method for nm-scale NDs from an organic precursor is encouraging^44^. Theoretical studies have differed in their conclusions as to whether sub-5nm NDs would support a stable substitutional boron state, offering semiconducting properties, or whether such a state would be unstable, with boron instead aggregating or attaching to edge structures^18–20^. In the present study detonation-derived NDs with purposefully added boron during the detonation process have been studied with a wide range of experimental techniques. The individual DNDs are of ~4 nm in size, and have been studied with CL, PL, Raman and IR spectroscopies, AFM and HR-TEM as well as electrically measured through the use of impedance spectroscopy. When the results from these differing techniques are combined it is apparent that the B-DNDs studied here do indeed support substitutional boron species and hence will be acting as semiconducting diamond nanoparticles. Evidence for moderate doping levels in some particles (~10^17^ B cm^−3^), is found alongside the observation that some particles are heavily doped (~10^20^ B cm^−3^) and likely to be quasi-metallic in character. Whilst further B-DND research is required to generate a more homogeneous distribution of doping concentration, the current study has shown that substitutional boron doping in sub-nm NDs is in fact possible, opening up the path to a whole host of new applications for this interesting class of nano-particles.

## Methods

### Detonation Nanodiamond

Boron-included DNDs were sourced from SkySpring Nanomaterials, and classified according to the data-sheet as 3–4 nm in size. The dry powder was placed in DI water at a concentration of 0.05 g/l, then ultra-sonicated on 40% power for 5 hours (Sonics Vibracell VCX 500; 500 W, 20 kHz). The solution was used to seed the DNDs on either RCA cleaned silicon substrates or H-terminated silicon substrates (microwave hydrogen plasma, Seki Technotron, AX1010, 1 kW, 20 mbar, 800 C, 20 minutes). Seeding was by immersion in an ultra-sonic bath for 3 minutes. In other cases the dry powder was used to create solid round pellets (11 mm diameter, 3 mm thick) using a mechanical press (10 T weight).

### DND treatments

“As-received” B-DNDs were treated with ozone to produce O-terminated DNDs (ozone, 50 mbar, 200 C, 3 hours) or hydrogen to generate H-terminated DNDs (H_2_ atmosphere at 10 mbar, 700 C, 5 hours).

### Characterization

Fourier transform infra-red (FTIR) spectroscopy was performed using a Perkin Elmer Spectrum One instrument that could be fitted with an ATR accessory. All experiments were preformed in a dry nitrogen atmosphere. Transmission FTIR involved mixing DNDs with KBr and pressing a pellet as described above. In the case of ATR-FTIR and thin layer of DNDs was deposited by sonication on CaF_2_ IR windows. Impedance Spectroscopy (IS) was performed in a stainless steel vacuum vessel in the temperature range 20–500 C, over the frequency range 0.1 Hz-10 MHz (Solartron 1260 Impedance system with Solartron 1296 Dielectric Interface). In a typical IS analysis, impedance is measured as a function of frequency, and the real component plotted against the imaginary component as the frequency is changed. These so-called ‘Cole-Cole’ plots may then reveal differing semicircular responses for each RC component of the film; repeating the measurements at differing temperatures enables the activation energy for each component to be determined. High resolution TEM was performed using a Joel-2100 instrument (200 kV); DND solutions were used to attach DNDs to 3 mm carbon coated Cu TEM grids by sonication. The HR-TEM used is capable of resolving lattice spacing as small as 0.14 nm. The same instrument was used for cathodoluminescence (CL) at low temperature (95 K) and beam energy (100 kV). AFM measurements (Bruker Icon, tapping mode) were performed on DND seeded Si substrates. Raman spectroscopy (Renishaw Invia, 532 nm, 150 mW) was used to reveal peaks characteristic of diamond and graphite phases.
